# Supplementary material for: Multiple hydrogen-bonding induced nonconventional red fluorescence emission in hydrogels
Source: Nat Commun. 2024 Apr 25;15:3482. doi: 10.1038/s41467-024-47880-7 (PMC11045767; doi:10.1038/s41467-024-47880-7)
Supplement: Supplementary file 1 — Supplementary Information [file 41467_2024_47880_MOESM1_ESM.pdf]

**Supplementary information**

# Multiple hydrogen-bonding induced nonconventional red fluorescence emission in hydrogels

*Jiayu Wu[1,2], Yuhuan Wang[3], Pan Jiang[1]*, Xiaolong Wang[1]*, Xin Jia[2], Feng Zhou[1]*

**1** State Key Laboratory of Solid Lubrication, Lanzhou Institute of Chemical Physics, Chinese Academy of Sciences, Lanzhou 730000, China.
**2** School of Chemistry and Chemical Engineering/State Key Laboratory Incubation Base for Green Processing of Chemical Engineering, Shihezi University, Shihezi 832003, China.
**3** School of Chemical Sciences, University of Chinese Academy of Sciences, Beijing 100049, China.
Corresponding authors: pan.jiang@ijm.fr, wangxl@licp.cas.cn

**This PDF file includes:**

Supplementary Methods

Supplementary Discussion

Supplementary Fig. 1 to 31

Supplementary Table 1 to 3

Supplementary References

## Supplementary Methods

### Materials

Acryloyl chloride (98%, Aladdin, Shanghai, China), Semicarbazide hydrochloride (98%, Acmec, Shanghai, China), diethyl ether (99.5%, Kelong Company, China), potassium carbonate anhydrous ($K_2CO_3$, 99%, Aladdin, Shanghai, China), Lithium phenyl-2,4,6-trimethylbenzoylphosphinate (LAP), Pancreatin (Biosharp, 143188), CCK-8 (Invigentech, IV08-100), PBS (Procell, WH0112201 911XP), MEM medium (Sebacon, iCell-0012). Deionized (DI) water was made in the laboratory.

### Structured fluorescence hydrogel printing

The hydrogel structure was fabricated by DLP 3D printing using hydrogel ink consisted of monomer NASC and photo-initiator LAP. The monomer NASC were dissolved in the mixed solvent of DMSO and DI (7/3, wt/wt), then LAP (0.3 wt% of the monomers) was added to mix by magnetic stirring at nitrogen atmosphere. The hydrogel structure was manufactured by a homemade DLP 3D printer with a 405 nm DMD. The curing time of each layer (5 s), light source (2K resolution, 1 W power) and layer thickness (0.1 mm) remained constant for all experiments. The as-prepared structured hydrogels were thoroughly immersed in DI water at 100 °C for 48 h. In this case, to further improve print accuracy, a small amount of photo-absorber (0.25 g/L tartrazine) was added to the above hydrogel ink for printing of the hydrogel castle and the Eiffel Tower structure (Fig. 5c).

### Light source information for taking photos

The fluorescent photographs of hydrogel were triggered by UV light source with photo power of 20 W in Fig. 1, Fig. 2, Fig. 3, Fig. 5, Fig. 6g, Supplementary Fig. 5, Supplementary Fig. 7, Supplementary Fig. 18, Supplementary Fig. 26, Supplementary Fig. 27, and Supplementary Fig. 29. The fluorescent photographs in Fig. 6b, c, e, and Supplementary Fig. 31 were taken under a 50 W UV light source. The wavelength of the light source is 365 nm.

**Supplementary Discussion**

**Chemical structure characterization**

As shown in Supplementary Fig. 2, the characteristic peaks of NASC monomers have been marked in the picture: δ = 9.8 ($H_a$), 7.9 ($H_b$), 6.2 ($H_c$), 5.9 ($H_d$), 6.1 and 5.7 ($H_e$). As shown in Supplementary Fig. 3, The characteristic peaks of **PNASC**$_{25°C}$, **PNASC**$_{45°C}$, **PNASC**$_{65°C}$, **PNASC**$_{85°C}$, and **PNASC**$_{100°C}$: 3340 (NH), 3185 (NH), 3033 (NH). Supplementary Fig. 4 shows the $^{13}C$ NMR spectrum of **PNASC**$_{25°C}$ and **PNASC**$_{100°C}$, respectively. The corresponding position has been marked in the picture. The results showed that the chemical structure of these hydrogels did not change.

**Relationship between polymer conformation and properties**

As shown in Supplementary Fig. 11 and Supplementary Fig. 12, NASC monomers are easy to form multi-molecule bonds when dissolved in pure DI, due to their urea group having the ability to form strong hydrogen bonds. The polymer chain segment clusters gather together after photopolymerization, which leads to severe phase separation (Supplementary Fig. 15); when DMSO with strong de-hydrogen bonding ability is added to the solution, NASC monomer can be well dispersed in the solution. The polymer network can be well stretched in the network of the hydrogel after photopolymerization. Then the cured hydrogel is soaked in deionized water, and the urea group between the polymer chains in the hydrogel forms strong interactions through hydrogen bonds after the leave of DMSO. The results showed that the highest density of hydrogen bonds formed between the polymer chains was observed when the DMSO content was 70 wt% of the total solvent content.

**Simulation calculation**

All-electron DFT calculations have been carried out by the latest version of ORCA quantum chemistry software[1] (Version 5.0.1). The BLYP functional was adopted for all calculations. For geometry optimization calculations, the def2-TZVP basis set[2] was used, and the optimal geometry for each compound was determined. The singlet point energy calculations were performed with B3LYP functional and ma-def2-TZVPP basis sets.[3] The SMD implicit solvation model[4] was used to account for the solvation effect of methanol. The DFT-D3 dispersion correction with BJ-damping[5,6] was applied to correct the weak interaction to improve the calculation accuracy. The Gibbs free energy change and the

energy of the HOMO-LUMO gap of $\mathbf{PNASC_{25°C}}$ and $\mathbf{PNASC_{100°C}}$ was shown in Supplementary Table 1 and Table 2.

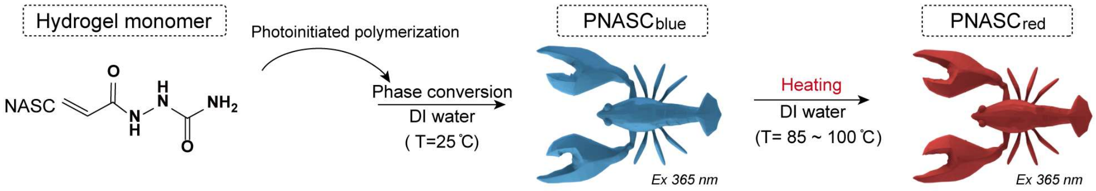


**Supplementary Fig. 1** | The preparation process of the fluorescent hydrogel.

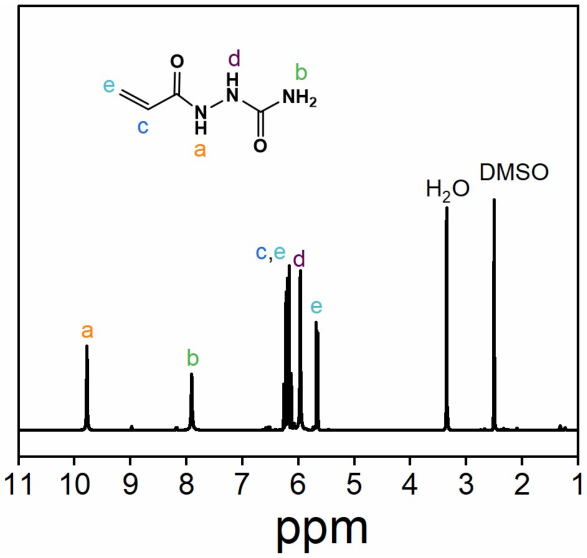

**Supplementary Fig. 2** | $^{1}$H NMR spectra of NASC monomers.

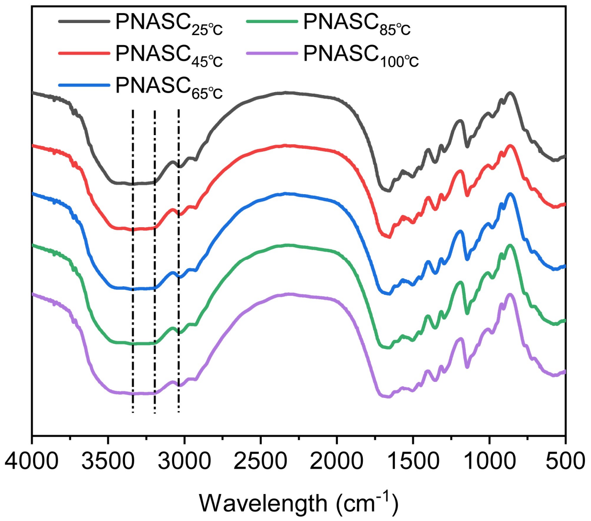


**Supplementary Fig. 3** | FTIR spectra of **PNASC$_{25℃}$**, **PNASC$_{45℃}$**, **PNASC$_{65℃}$**, **PNASC$_{85℃}$**, and **PNASC$_{100℃}$**.

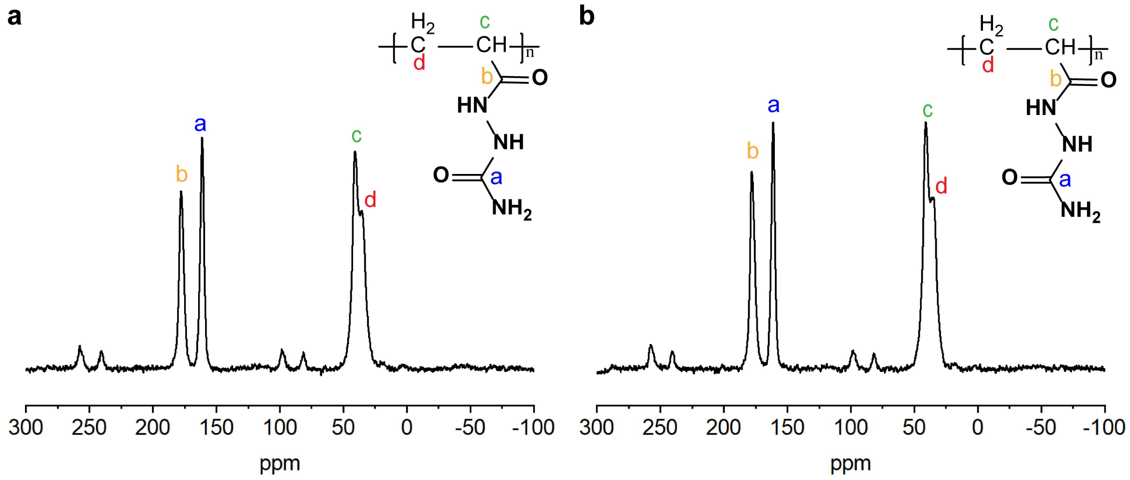


**Supplementary Fig. 4 | a**, $^{13}$C NMR spectra of **PNASC$_{25°C}$**. **b**, $^{13}$C NMR spectra of **PNASC$_{100°C}$**.

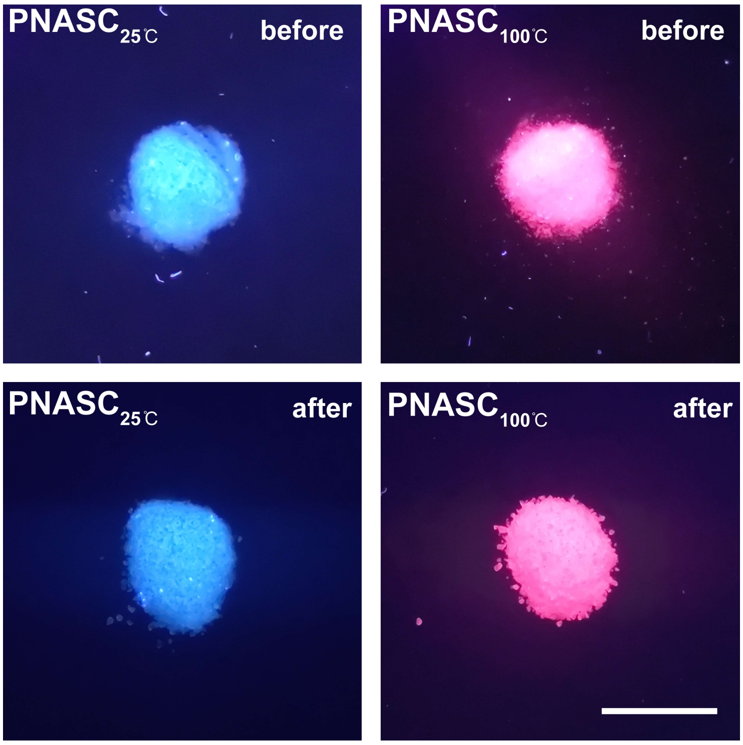


**Supplementary Fig. 5** | The hydrogels powder images of **PNASC$_{25℃}$** and **PNASC$_{100℃}$** under $E_x$ 365 nm. Before and after heating in 200 °C oven for 8 hours. The scale bar is 1 cm.

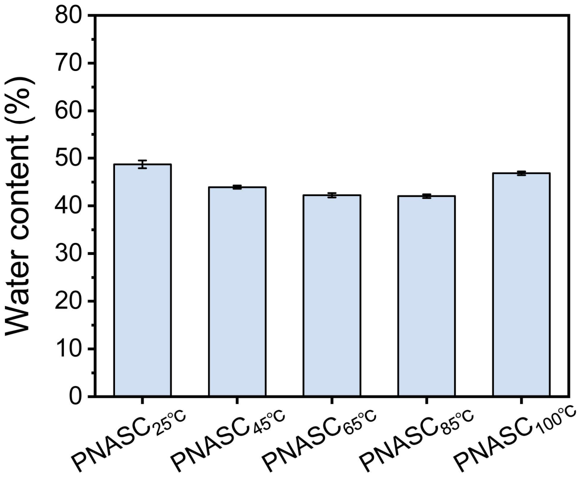


**Supplementary Fig. 6 |** The water content of **PNASC$_{25°C}$**, **PNASC$_{45°C}$**, **PNASC$_{65°C}$**, **PNASC$_{85°C}$**, and **PNASC$_{100°C}$**. Error bars represent the standard deviation (n = 3). The data is presented as mean values ± SD.

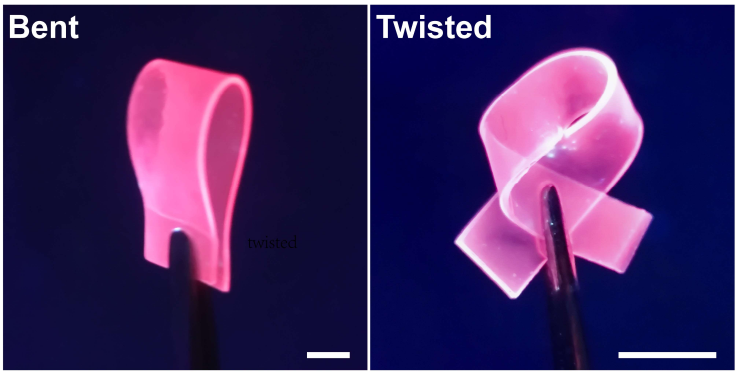


**Supplementary Fig. 7** | Digital photos of the **PNASC$_{100°C}$** hydrogel with the different shapes under UV light (365 nm). The scale bar is 5 mm.

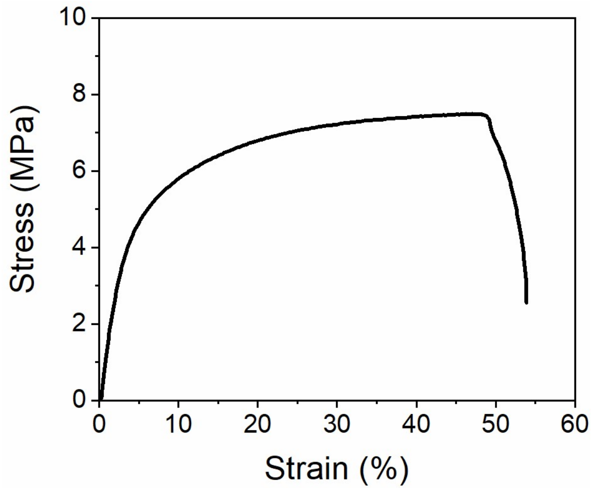


**Supplementary Fig. 8 |** Tensile-strain curves of **PNASC$_{100℃}$**.

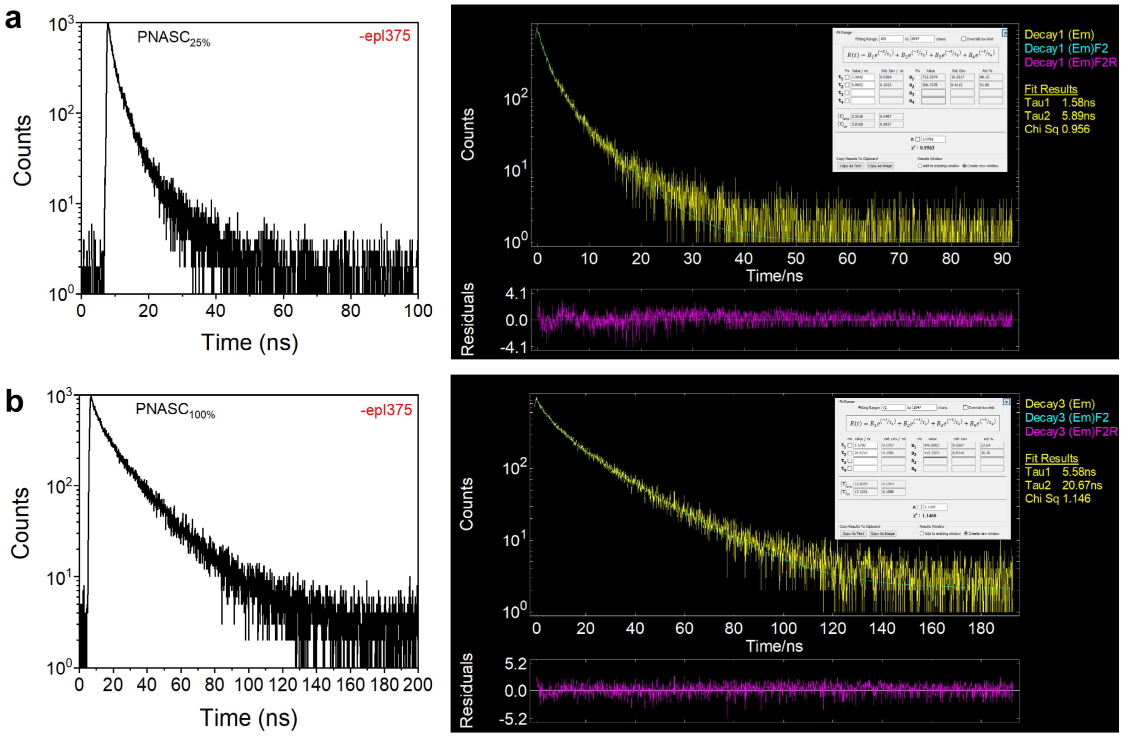


**Supplementary Fig. 9** | Time-resolved fluorescence decay curves of hydrogels. (a) The decay curves of **PNASC$_{25°C}$** and corresponding fitted data. (b) The decay curves of **PNASC$_{100°C}$** and corresponding fitted data.

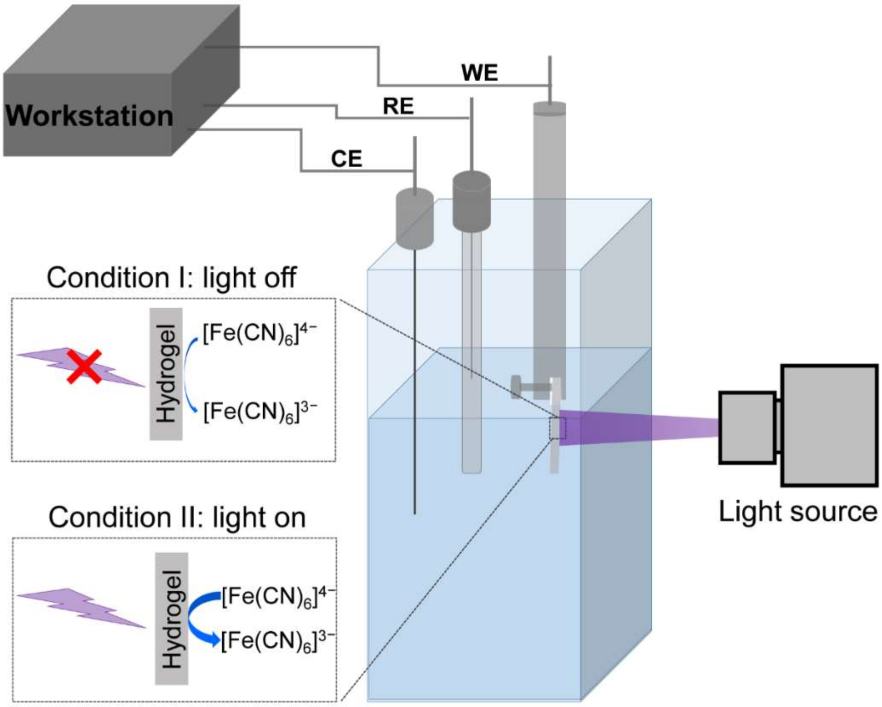


**Supplementary Fig. 10** | Schematic diagram of the electrochemical testing process.

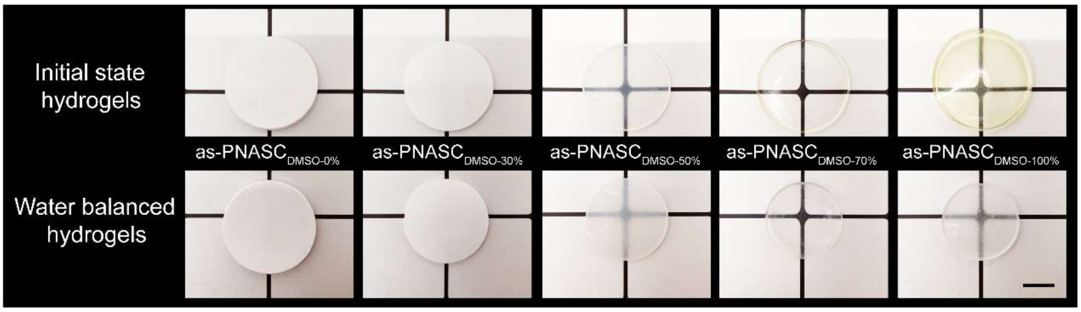


**Supplementary Fig. 11** | Digital photos of hydrogels before and after equilibrium in water. The scale bar is 1 cm.

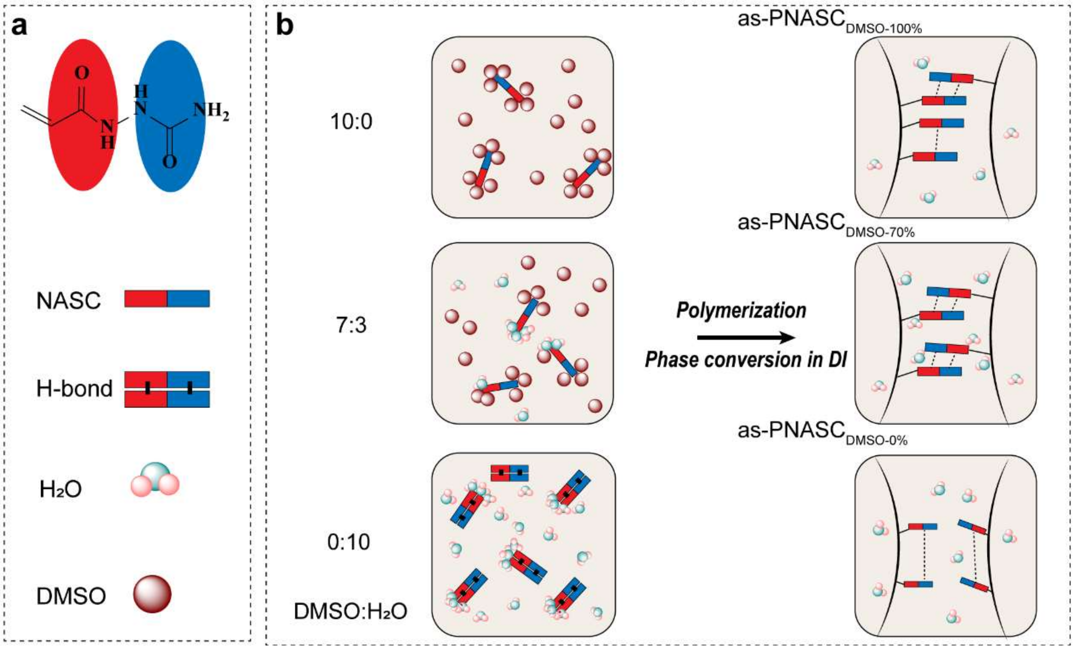


**Supplementary Fig. 12** | **a**, Main components of hydrogel ink. **b**, Schematic representation of the conformation of the polymer network in **as-PNASC$_{DMSO-0\%}$**, **as-PNASC$_{DMSO-70\%}$**, and **as-PNASC$_{DMSO-100\%}$**.

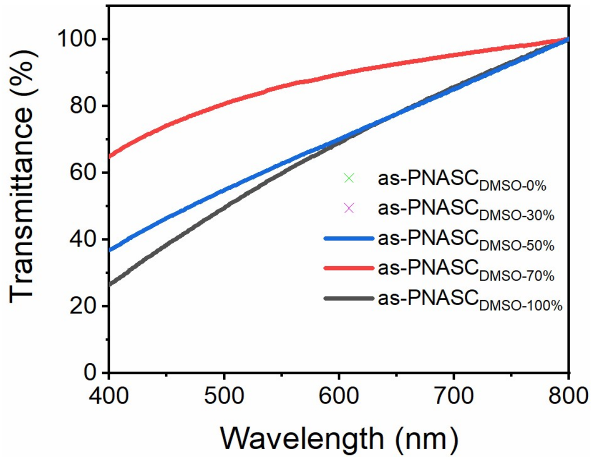


**Supplementary Fig. 13** | The transmittance of **as-PNASC$_{DMSO-0\%}$**, **as-PNASC$_{DMSO-30\%}$**, **as-PNASC$_{DMSO-50\%}$**, **as-PNASC$_{DMSO-70\%}$**, and **as-PNASC$_{DMSO-100\%}$**.

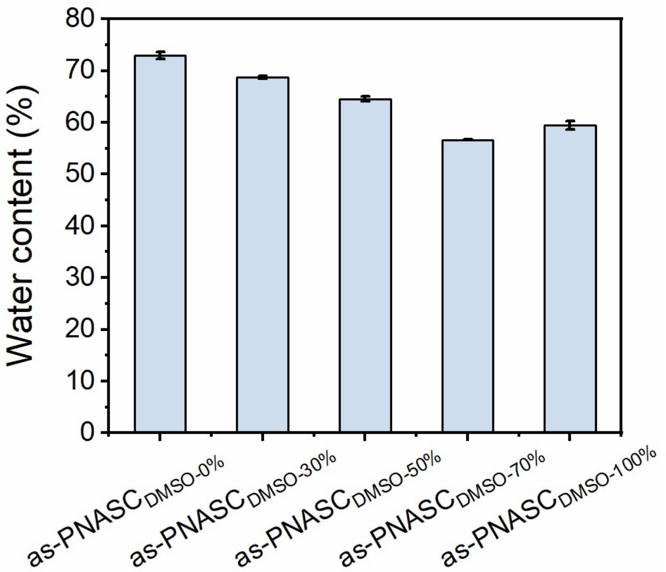


**Supplementary Fig. 14** | The water content of **as-PNASC$_{\mathbf{DMSO\text{-}0\%}}$**, **as-PNASC$_{\mathbf{DMSO\text{-}30\%}}$**, **as-PNASC$_{\mathbf{DMSO\text{-}50\%}}$**, **as-PNASC$_{\mathbf{DMSO\text{-}70\%}}$**, and **as-PNASC$_{\mathbf{DMSO\text{-}100\%}}$**. Error bars represent the standard deviation (n = 3). The data is presented as mean values ± SD.

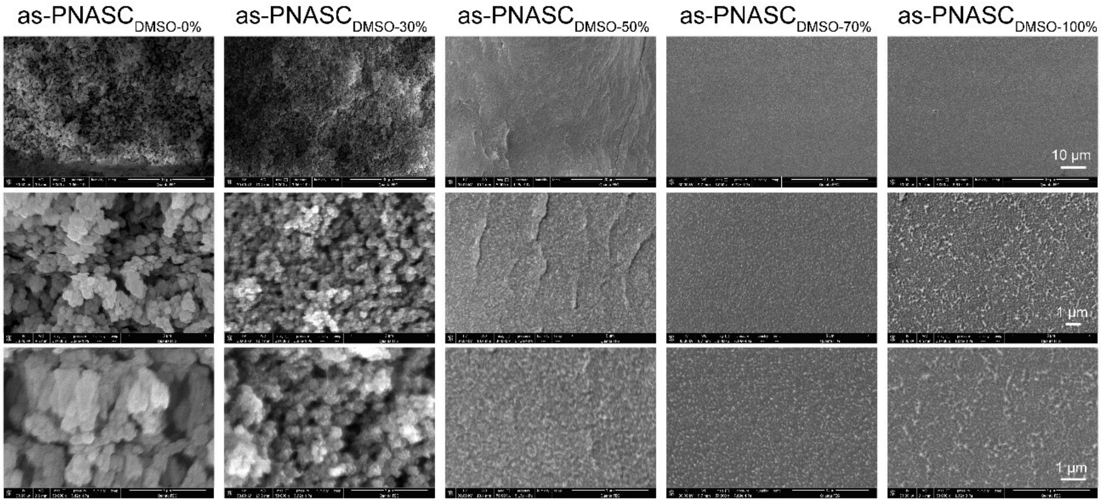


**Supplementary Fig. 15 |** The cross-section SEM images of **as-PNASC$_{\mathbf{DMSO\text{-}0\%}}$**, **as-PNASC$_{\mathbf{DMSO\text{-}30\%}}$**, **as-PNASC$_{\mathbf{DMSO\text{-}50\%}}$**, **as-PNASC$_{\mathbf{DMSO\text{-}70\%}}$**, and **as-PNASC$_{\mathbf{DMSO\text{-}100\%}}$**.

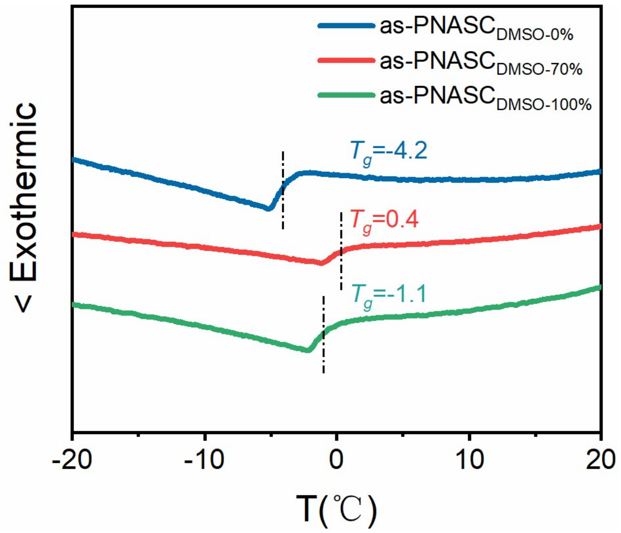


**Supplementary Fig. 16** | DSC thermograms of the powder of **as-PNASC$_{\mathbf{DMSO\text{-}0\%}}$**, **as-PNASC$_{\mathbf{DMSO\text{-}70\%}}$**, and **as-PNASC$_{\mathbf{DMSO\text{-}100\%}}$**.

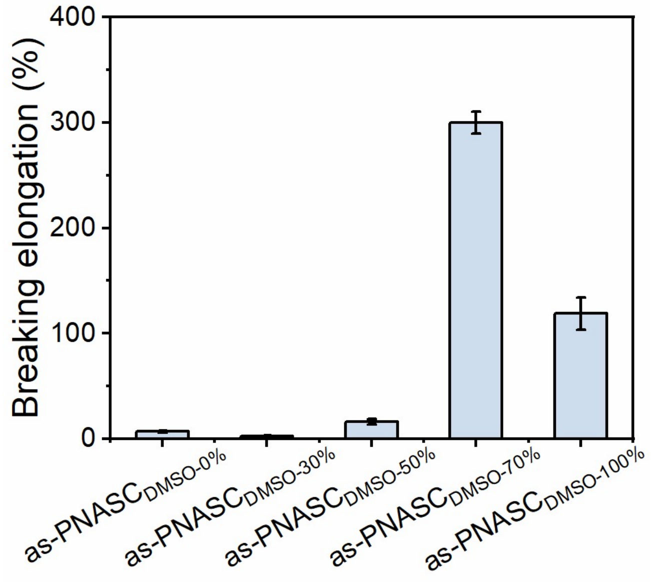


**Supplementary Fig. 17** | Breaking elongation **as-PNASC$_{DMSO-0\%}$**, **as-PNASC$_{DMSO-30\%}$**, **as-PNASC$_{DMSO-50\%}$**, **as-PNASC$_{DMSO-70\%}$**, and **as-PNASC$_{DMSO-100\%}$**. Error bars represent the standard deviation (n = 3). The data is presented as mean values ± SD.

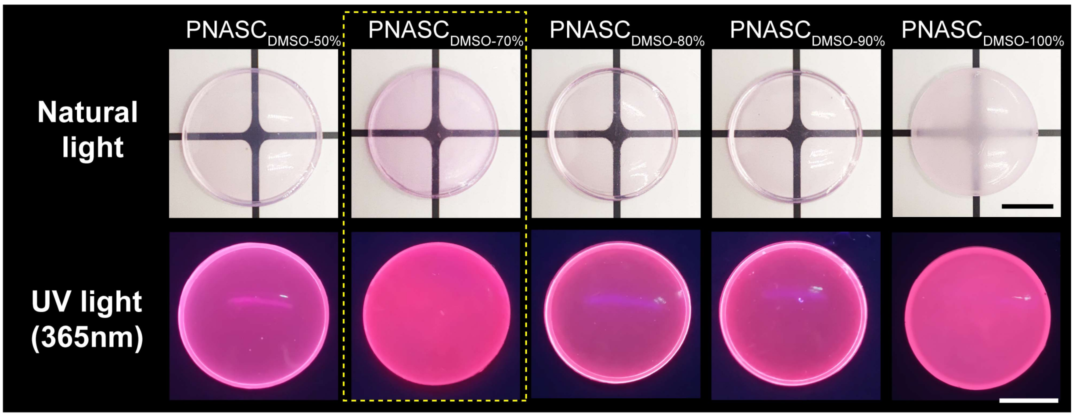


**Supplementary Fig. 18** | Digital photos of **PNASC$_{DMSO-50\%}$**, **PNASC$_{DMSO-70\%}$**, **PNASC$_{DMSO-80\%}$**, **PNASC$_{DMSO-90\%}$**, and **PNASC$_{DMSO-100\%}$** under natural light and UV light (365 nm), The scale bar is 1 cm.

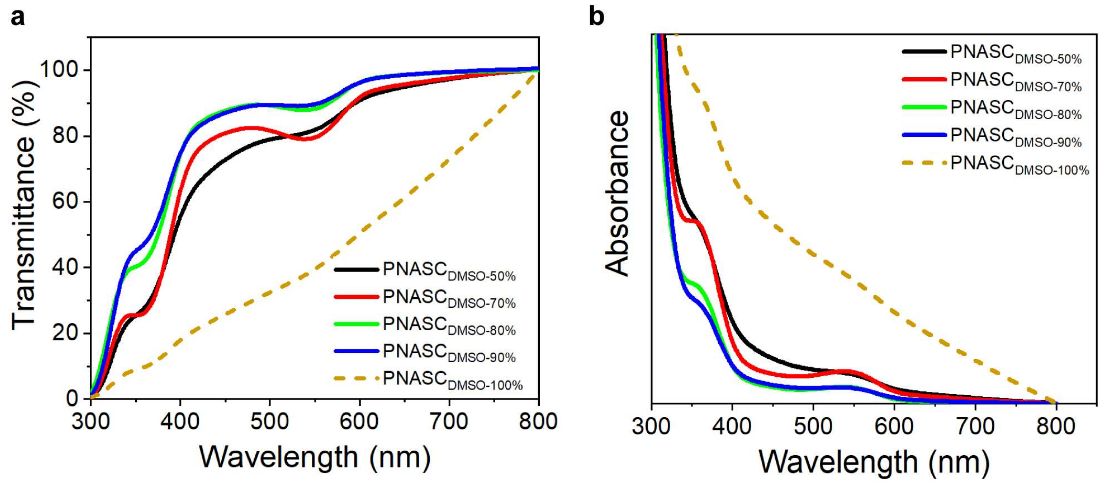


**Supplementary Fig. 19** | **a**, Transmittance and (**b**) absorbance images of **PNASC$_{DMSO-50\%}$**, **PNASC$_{DMSO-70\%}$**, **PNASC$_{DMSO-80\%}$**, **PNASC$_{DMSO-90\%}$**, and **PNASC$_{DMSO-100\%}$**.

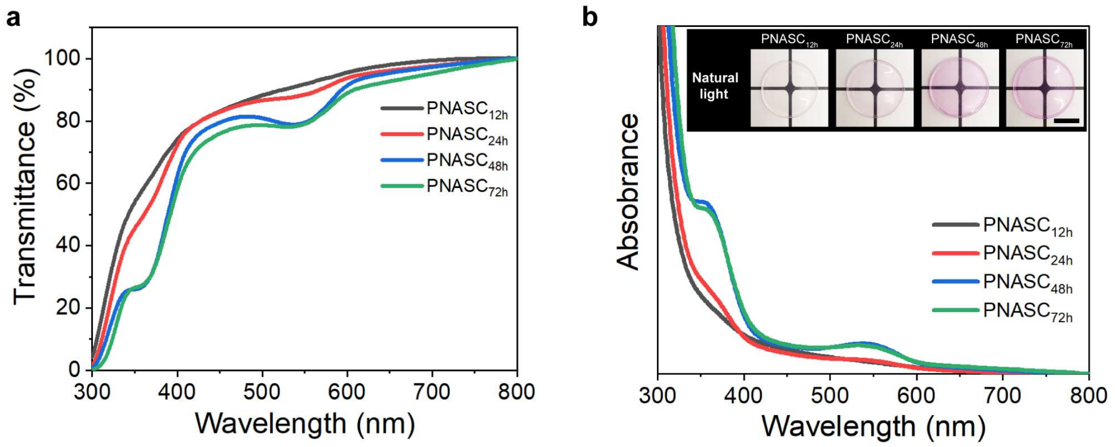


**Supplementary Fig. 20 | a**, Transmittance and (**b**) absorbance images of **PNASC$_{12h}$**, **PNASC$_{24h}$**, **PNASC$_{48h}$**, and **PNASC$_{72h}$**. The inset is digital photos of hydrogels under natural light. The scale bar is 1 cm.

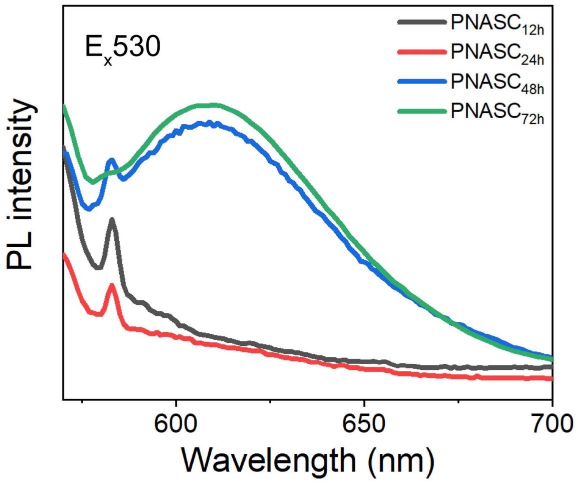


**Supplementary Fig. 21** | Emission spectra of **$PNASC_{12h}$**, **$PNASC_{24h}$**, **$PNASC_{48h}$**, and **$PNASC_{72h}$**. Excited at 530 nm.

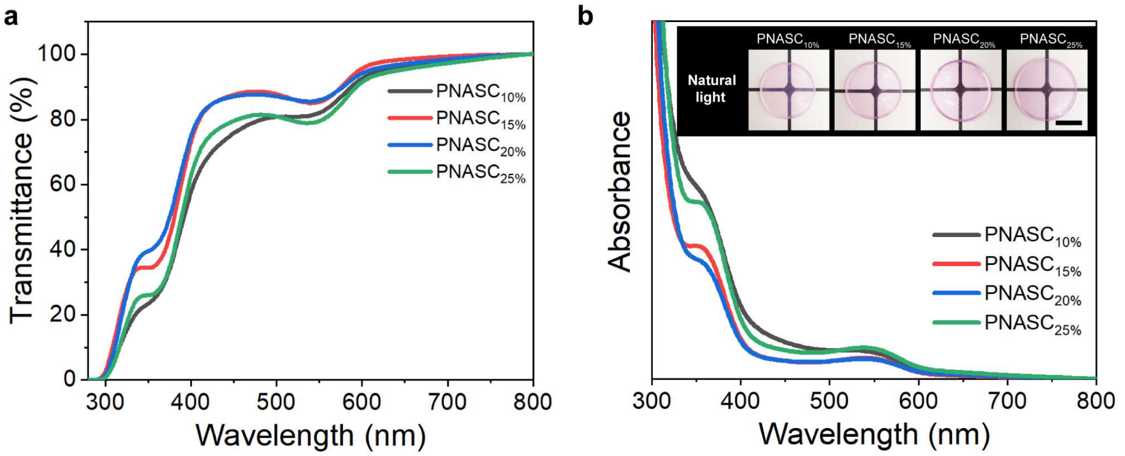


**Supplementary Fig. 22 | a**, Transmittance and (**b**) absorbance images of **PNASC$_{10\%}$**, **PNASC$_{15\%}$**, **PNASC$_{20\%}$**, and **PNASC$_{25\%}$**. The inset is digital photos of hydrogels under natural light. The scale bar is 1 cm.

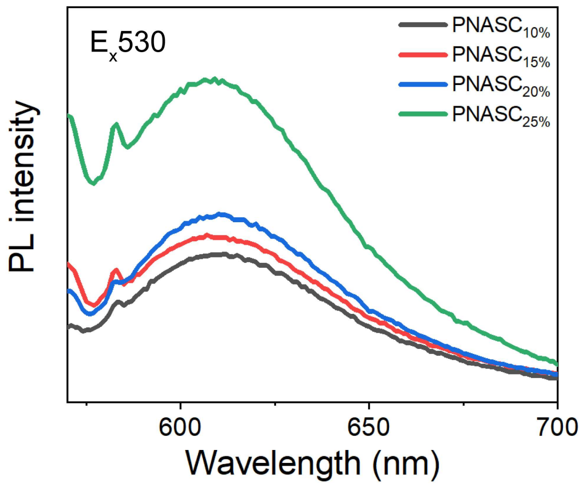


**Supplementary Fig. 23** | Emission spectra of **PNASC$_{10\%}$**, **PNASC$_{15\%}$**, **PNASC$_{20\%}$**, and **PNASC$_{25\%}$**. Excited at 530 nm.

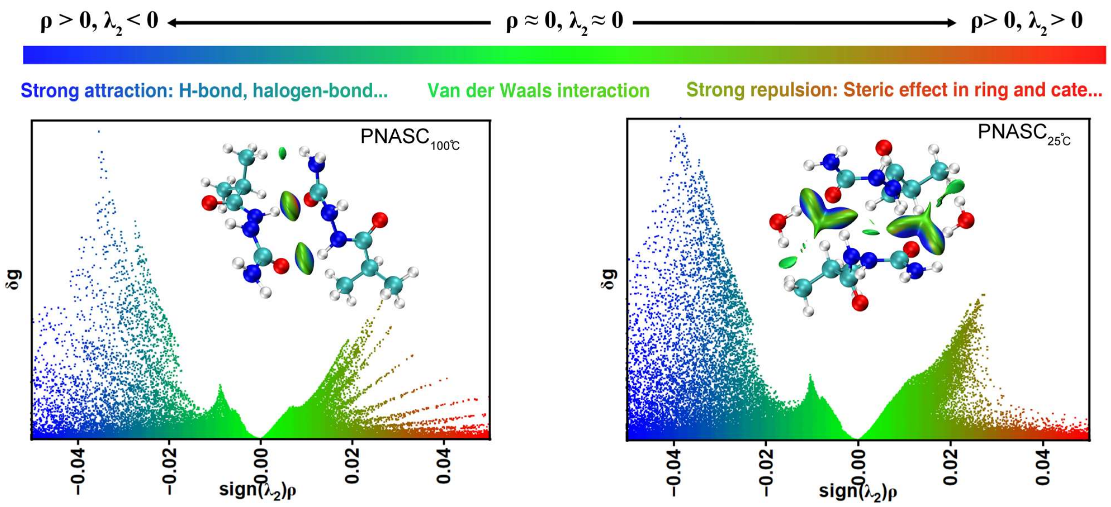


**Supplementary Fig. 24** | Scatter graphs and isosurfaces of the noncovalent interactions of **PNASC$_{25\%}$** and **PNASC$_{100\%}$** by IGM method (Blue and green indicate strong and weak attraction, respectively. Red means repulsion). **PNASC$_{100℃}$** hydrogel has a clear formation of two hydrogen bonds with a blue color in the middle of the two isosurfaces. It can be seen in the scatter plot, there is a peak in the blue region corresponding to the formation of a hydrogen bond. However, **PNASC$_{25℃}$** state not only has two hydrogen bonds formed by -C=O and -NH, but also two strong isosurfaces with water molecules. The results show that the formed H-bonds between -C=O and -N-H are harassed by hydrated water molecules in the hydrogel matrix, which results in an inhibited interchain hydrogen bonding and less possibility to achieve delocalization of H-bond D-A.

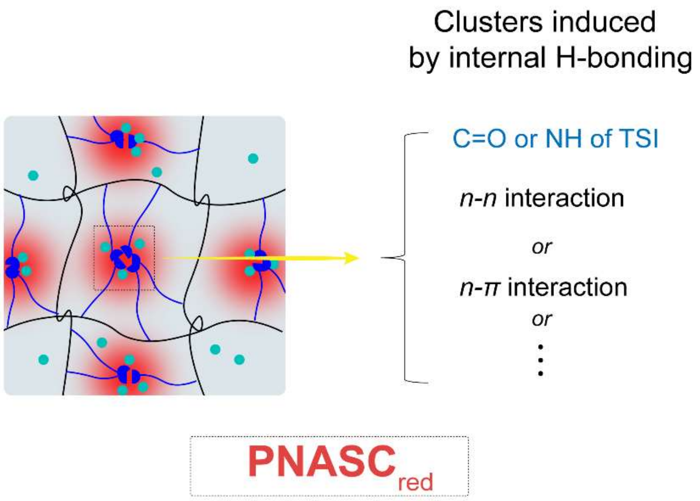


**Supplementary Fig. 25 |** Interaction of cluster luminescence in **PNASC$_{red}$** hydrogels.

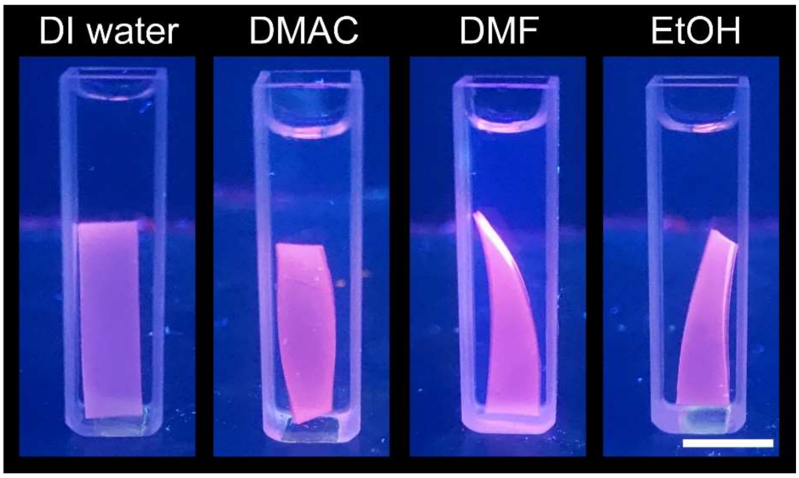


**Supplementary Fig. 26** | Photos of **PNASC$_{100℃}$** hydrogel in DI water, DMAC, DMF, and EtOH, respectively. Under UV light (365 nm). The scale bar is 1 cm.

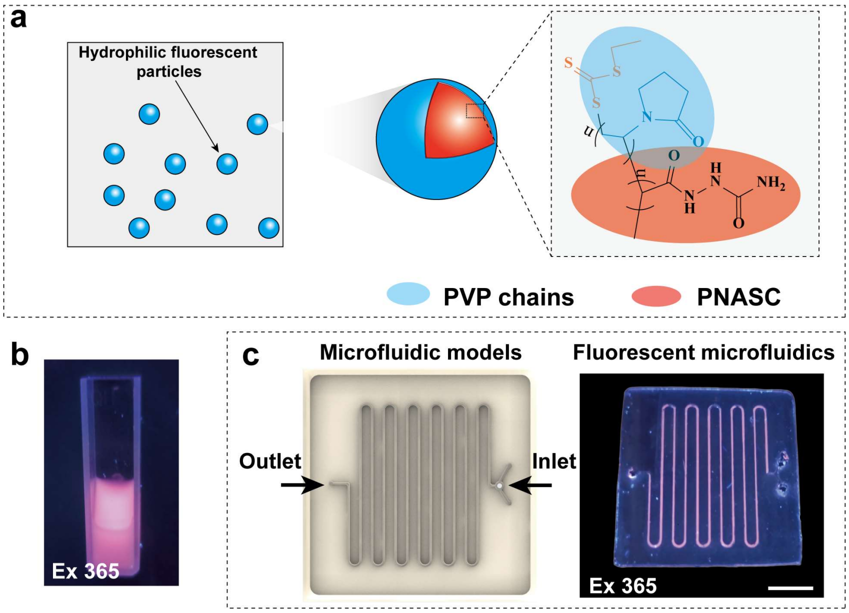


**Supplementary Fig. 27 | a**, Schematic diagram of the composition of hydrophilic fluorescent particles. **b**, Photograph of aqueous solution of fluorescent polymer particles. **c**, Microfluidic model and photos of fluorescent particles in aqueous solution. The scale bar is 1 cm.

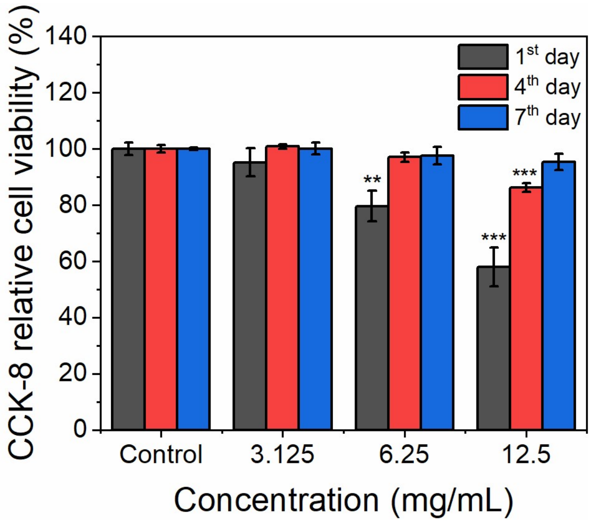


**Supplementary Fig. 28** | CCK-8 assay of cell viability. n=3, α=0.05, *p < 0.05, **p < 0.01, ***p < 0.001, two-tailed t-test. Error bars represent the standard deviation (n = 3). The data is presented as mean values ± SD.

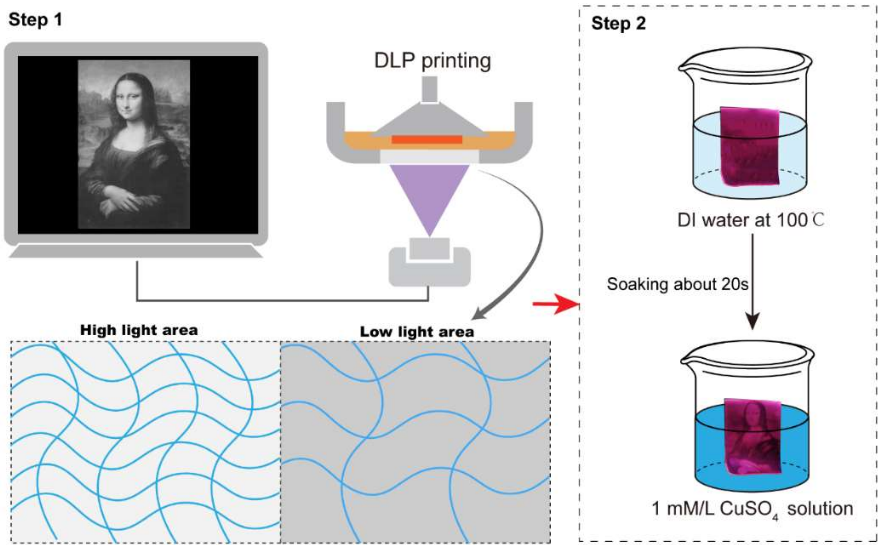


**Supplementary Fig. 29** | Schematic diagram of the preparation process of hydrogels with grayscale fluorescence pattern.

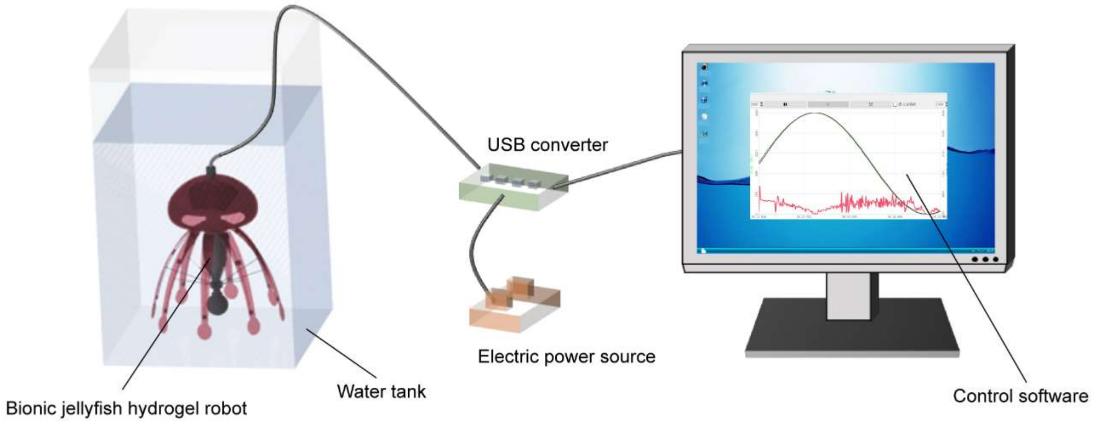


**Supplementary Fig. 30 |** Schematic diagram of the device of a bionic jellyfish hydrogel robot.

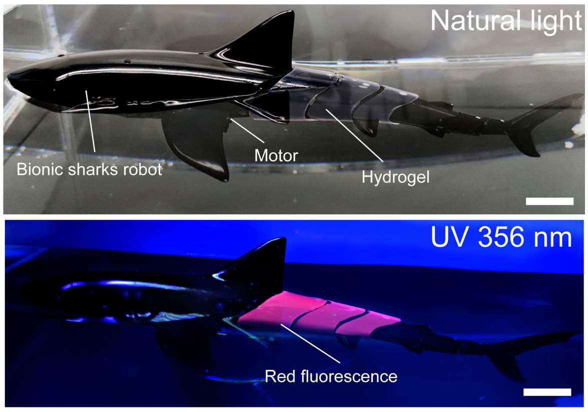


**Supplementary Fig. 31** | Digital photographs of the bionic shark robot under natural and UV 365 nm light. The scale bar is 3 cm.

**Supplementary Table 1** | The Gibbs free energy change of **$PNASC_{25°C}$** and **$PNASC_{100°C}$** at 100°C.

| | **Gcorr** | **E** | **G** | **ΔG** | **kcal/mol** | **kJ/mol** | **eV** |
|---|---|---|---|---|---|---|---|
| Mol | 0.119131 | -511.866 | -511.746 | | | | |
| Water | -0.00295 | -76.4441 | -76.447 | | | | |
| $PNASC_{25°C}$ | 0.305782 | -1176.66 | -1176.35 | -0.024 | -15.060 | -63.012 | -0.653 |
| $PNASC_{100°C}$ | 0.266039 | -1023.74 | -1023.48 | | | | |

**Supplementary Table 2** | The energy of HOMO-LUMO gap of **$PNASC_{25°C}$** and **$PNASC_{100°C}$**.

| | Orbital | Energy | | HOMO-LUMO gap | | |
|---|---|---|---|---|---|---|
| $PNASC_{25°C}$ | HOMO | -0.270844 a.u. | -7.370052 eV | 0.260914 a.u. | 7.099828 eV | 685.029436 kJ/mol |
| | LUMO | -0.009931 a.u. | -0.270224 eV | | | |
| $PNASC_{100°C}$ | HOMO | -0.244771 a.u. | -6.660560 eV | 0.236447 a.u. | 6.434057 eV | 620.792241 kJ/mol |
| | LUMO | -0.008324 a.u. | -0.226503 eV | | | |

**Supplementary Table 3** | Information on NCTC clone 929 [L cell, L-929]

| | |
|---|---|
| **Cell name** | NCTC clone 929 [L cell, L-929] |
| **Cell Source** | Procell Life Science & Technology Co., Ltd. |
| **Item No.** | CL-0137 |
| **Date of testing** | 26th May 2022 |

**STR identification**

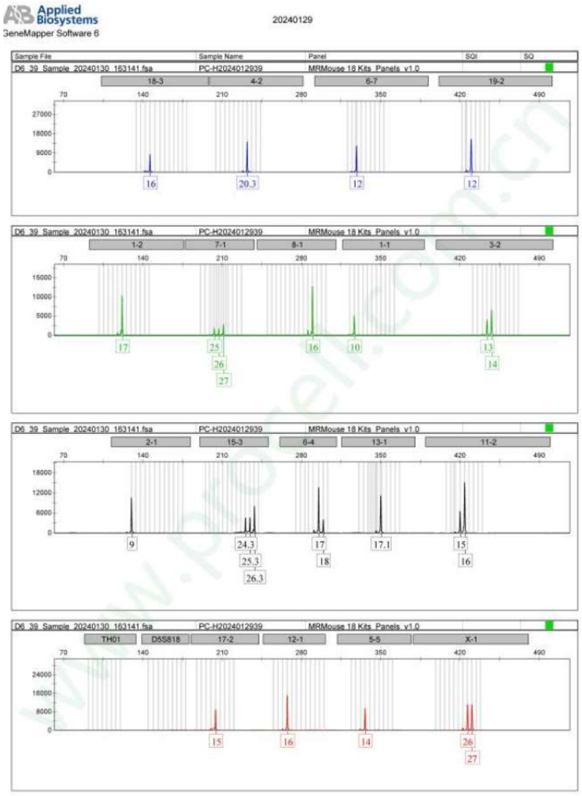

## Supplementary References


1 Neese, F. Software update: the ORCA program system, version 4.0. *WIREs Mol. Sci.* **8**, e1327 (2017).

2 Weigend, F. & Ahlrichs, R. Balanced basis sets of split valence, triple zeta valence and quadruple zeta valence quality for H to Rn: Design and assessment of accuracy. *Phys. Chem. Chem. Phys.* **7**, 3297-3305 (2005).

3 Zheng, J., Xu, X. & Truhlar, D. G. Minimally augmented Karlsruhe basis sets. *Theor. Chem. Acc.* **128**, 295-305 (2010).

4 Marenich, A. V., Cramer, C. J. & Truhlar, D. G. Universal solvation model based on solute eelectron density and on a continuum model of the solvent defined by the bulk dielectric constant and atomic surface tensions. *J. Phys. Chem. B* **113**, 6378-6396 (2009).

5 Grimme, S., Ehrlich, S. & Goerigk, L. Effect of the damping function in dispersion corrected density functional theory. *J. Comput. Chem.* **32**, 1456-1465 (2011).

6 Grimme, S., Antony, J., Ehrlich, S. & Krieg, H. A consistent and accurate ab initio parametrization of density functional dispersion correction (DFT-D) for the 94 elements H-Pu. *J. Chem. Phys.* **132**, 154104 (2010).
