# Supplementary material for: Multiple hydrogen-bonding induced nonconventional red fluorescence emission in hydrogels
Source: Nat Commun. 2024 Apr 25;15:3482. doi: 10.1038/s41467-024-47880-7 (PMC11045767; doi:10.1038/s41467-024-47880-7)
Supplement: Supplementary file 3 — Description of Additional Supplementary Files [file 41467_2024_47880_MOESM3_ESM.pdf]

Description of Additional Supplementary Files

File Name: Supplementary Data 1
Description: Electronic structure calculations of the atomic coordinates of the optimized computational models for Lumo and Homo (1), and IGM (2).

File Name: Supplementary Movie 1
Description: Bent and folded of **$PNASC_{100℃}$** hydrogel. Bent and folded of **$PNASC_{100℃}$** hydrogels under 365 nm light demonstrated the excellent flexibility of the hydrogels.

File Name: Supplementary Movie 2
Description: Simulation animation of a bionic jellyfish hydrogel underwater robot motion.

File Name: Supplementary Movie 3
Description: **$PNASC_{100℃}$** hydrogel as fluorescent components for a bionic jellyfish underwater robot. Servomotor extending/contracting at a rate of 5.34 s/cycle.

File Name: Supplementary Movie 4
Description: Contaminant detecting process of the fluorescent electric sharks. The bionic robotic shark as a fluorescent sensing device equipped with our fluorescent hydrogel skin.
